# Autophagy induction by leptin contributes to suppression of apoptosis in cancer cells and xenograft model: Involvement of p53/FoxO3A axis

**DOI:** 10.18632/oncotarget.3347

**Published:** 2015-01-31

**Authors:** Saroj Nepal, Mi Jin Kim, Jin Tae Hong, Sang Hyun Kim, Dong-Hwan Sohn, Sung Hee Lee, Kyung Song, Dong Young Choi, Eung Seok Lee, Pil-Hoon Park

**Affiliations:** ^1^ College of Pharmacy, Yeungnam University, Gyeongsangbuk-do, Republic of Korea; ^2^ College of Pharmacy and Medical Research Center, Chungbuk National University, Cheongju, Chungbuk, Republic of Korea; ^3^ Department of Pharmacology, School of Medicine, Kyungpook National University, Daegu, Republic of Korea; ^4^ Institute of Pharmaceutical Research and Development, College of Pharmacy, Wonkwang University, Iksan, Jeonbuk, Republic of Korea

**Keywords:** apoptosis, autophagy, FoxO3A, leptin, p53

## Abstract

Leptin, a hormone mainly produced from adipose tissue, has been shown to induce proliferation of cancer cells. However, the molecular mechanisms underlying leptin-induced tumor progression have not been clearly elucidated. In the present study, we investigated the role of autophagy in leptin-induced cancer cell proliferation using human hepatoma (HepG2) and breast cancer cells (MCF-7), and tumor growth in a xenograft model. Herein, we showed that leptin treatment caused autophagy induction as assessed by increase in expression of autophagy-related genes, including beclin-1, Atg5 and LC3 II, further induction of autophagosome formation and autophagic flux. Interestingly, inhibition of autophagic process by treatment with inhibitors and LC3B gene silencing blocked leptin-induced increase in cell number and suppression of apoptosis, indicating a crucial role of autophagy in leptin-induced tumor progression. Moreover, gene silencing of p53 or FoxO3A prevented leptin-induced LC3 II protein expression, suggesting an involvement of p53/FoxO3A axis in leptin-induced autophagy activation. Leptin administration also accelerated tumor growth in BALB/c nude mice, which was found to be autophagy dependent. Taken together, our results demonstrate that leptin-induced tumor growth is mediated by autophagy induction and autophagic process would be a promising target to regulate development of cancer caused by leptin production.

## INTRODUCTION

Leptin, an adipokine secreted mainly from adipose tissue, is originally known to regulate appetite via acting on its receptors localized in the hypothalamic region. Following studies have established the cardinal role of leptin in the pathogenesis of obesity (controlling body fat mass) and diabetes [1]. Leptin produces various biological responses via activation of JAK/STAT pathway, mitogen-activated protein kinase and PI3 kinase pathways, all of which are well known to control cellular proliferation, differentiation and survival [2]. Compelling evidences have indicated that leptin stimulates growth of different types of cancer cells [3-6]. Leptin-induced tumor growth is mediated through suppression of apoptosis via down-regulating expression of apoptotic proteins, including Bax, in cancer cells [7], as well as stimulating cellular proliferation by activation of different types of kinase pathways [8-10]. Epidemiological studies have established obesity as an important risk factor for cancer development. One of the plausible mechanisms is the misbalance in plasma levels of adipokines. In particular, serum leptin level is increased, while adiponectin level is decreased in the obese [5, 11]. Although an increased leptin level is positively associated with obesity and cancer risk, detailed molecular mechanisms underlying are not clearly understood.

Autophagy, a cellular catabolic pathway responsible for the removal of faulty/unwanted cellular components via lysosomal degradation, has been shown to play an important role in the modulation of numerous pathophysiological processes. The autophagy is a complicated process requiring a number of autophagy-related genes (Atgs) to act at multiple stages of autophagy. In general, autophagic process usually involves initiation, nucleation and elongation step. During initiation, an isolation membrane is formed in the cytosol via class I PI3K activation and Atg complex involving uncoordinated 51-like kinase 1(ULK1), Atg13 and Atg17. This leads to activation of class III PI3K and nucleation with beclin-1. Finally, closure of isolation membrane, which is controlled by Atg5, Atg12, and LC3/Atg8, occurs [12, 13]. Autophagy induction in response to stressful stimuli is involved in adaptation of the cells to various stressful conditions, such as shortage of nutrients and oxygen [13], or it can also cause dysregulated cell death [14]. Cancer cells in the tumor tissue are typical example exposed to the stressful conditions and growing evidences have suggested the critical role of autophagy in the regulation and/or progression of cancer. The role of autophagy in cancer progression is controversial. For example, cancer cells activate autophagy in response to stressful insults, allowing cells to mitigate damage and promote senescence that limit tumorigenesis, indicating a regulatory role of autophagy in cancer development [15]. However, many other recent studies have indicated autophagy as a cytoprotective adaptive response. Activation of oncogenes and inactivation of tumor suppressor genes in cancer cells enhance autophagy, thereby promoting survival of tumor cells [16]. Furthermore, inhibition of autophagy by pharmacological (e.g. chloroquine or 3-methyladenine) or genetic interference (e.g., knocking down of essential proteins) accelerates cell death in conditions exposed to the cellular stress [16]. In addition, autophagy and apoptosis mutually negatively regulate each other. Autophagic process therefore contributes to the development of cancer [14, 17]. All these reports indicate that autophagy modulates cancer progression in a context-dependent manner. Detailed molecular mechanisms underlying what determines the differential role of autophagy in tumor growth remains to be further investigated.

p53 has been shown to induce autophagic process in response to cellular stresses in many cancer cell lines, in addition to its well-known tumor suppressing effects. For the induction of autophagic process, p53 induces expression of sestrins 1 and 2 genes, which activates AMPK [18]. AMPK activation in turn governs the activities of a number of transcription factors, including nuclear factor (erythroid-derived 2)-like 2(Nrf2) and the forkhead box O3A (FoxO3A), that induces expression of autophagy-related genes [19]. Interestingly, in contrast to the tumor suppressing effects, p53 signaling is also involved in cancer progression via increasing flux through pentose-phosphate pathway and shielding cancer cells from ROS mediated cell death, as well as promoting anabolism for further tumor cell growth [20]. In addition, recent studies have also demonstrated potential crosstalk between p53 and FoxO3A transcription factor, thereby modulating cell death or proliferation [21, 22].

Based on previous reports, it is well accepted that autophagy plays an important role in the development of cancer and leptin induces tumor growth. However, the effects of leptin on autophagy induction and its potential roles in cancer development have not been explored. Thus, to better understand the mechanisms underlying leptin-induced tumor growth, herein we investigated the potential role of autophagy in leptin-induced tumor growth and molecular mechanisms underlying autophagy induction. The present study provides the first evidence that leptin-induced autophagy is tightly linked with suppression of apoptosis. Moreover, we also uncovered that p53-FoxO3A axis plays a cardinal role in leptin-induced autophagy and suppression of apoptosis in cancer cells.

## RESULTS

### Leptin induces increase in cell number and suppresses apoptosis in cancer cells

**Figure 1:**
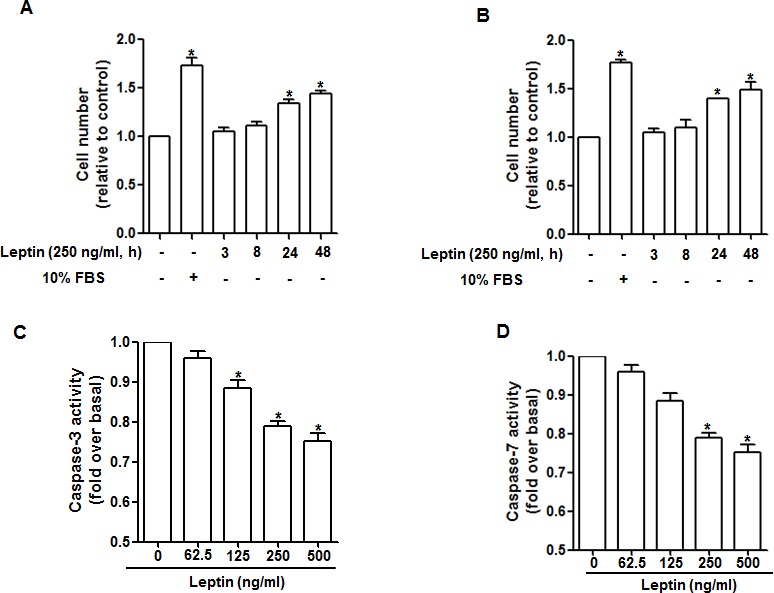
Effects of leptin on cell viability and apoptosis HepG2 cells (A) and MCF-7 cells (B) were treated with leptin for the indicated time points. Cell number was measured via MTS assay as described previously. HepG2 cells (C) and MCF-7 cells (D) were treated with the indicated concentrations of leptin for 48 h. Caspase-3/7 activity was determined as described in material and methods section. Values fold increase in comparison to cells not treated with leptin are presented as mean ± SEM (n=3). *P<0.05 compared with cells not treated with leptin.

To verify the effect of leptin on growth of cancer cells in our experimental conditions, we first investigated the effect of leptin on cell number using MTS assay. As expected, leptin increased cell number in a time dependent manner both in HepG2 (Fig. 1A) and MCF-7 (Fig. 1B) cells. We next assessed the effect of leptin on apoptosis via caspase-3/7 activity assay. Leptin suppressed caspase-3 activity dose dependently in HepG2 cells (Fig. 1C) and caspase-7 activity in MCF-7 cells (Fig. 1D). These results suggest that leptin treatment induces increase in hepatoma and breast cancer cells probably via suppression of apoptosis.

### Leptin induces autophagy in HepG2 and MCF-7 cells

**Figure 2:**
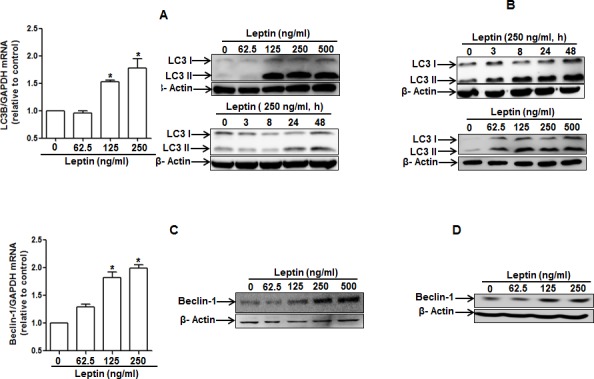

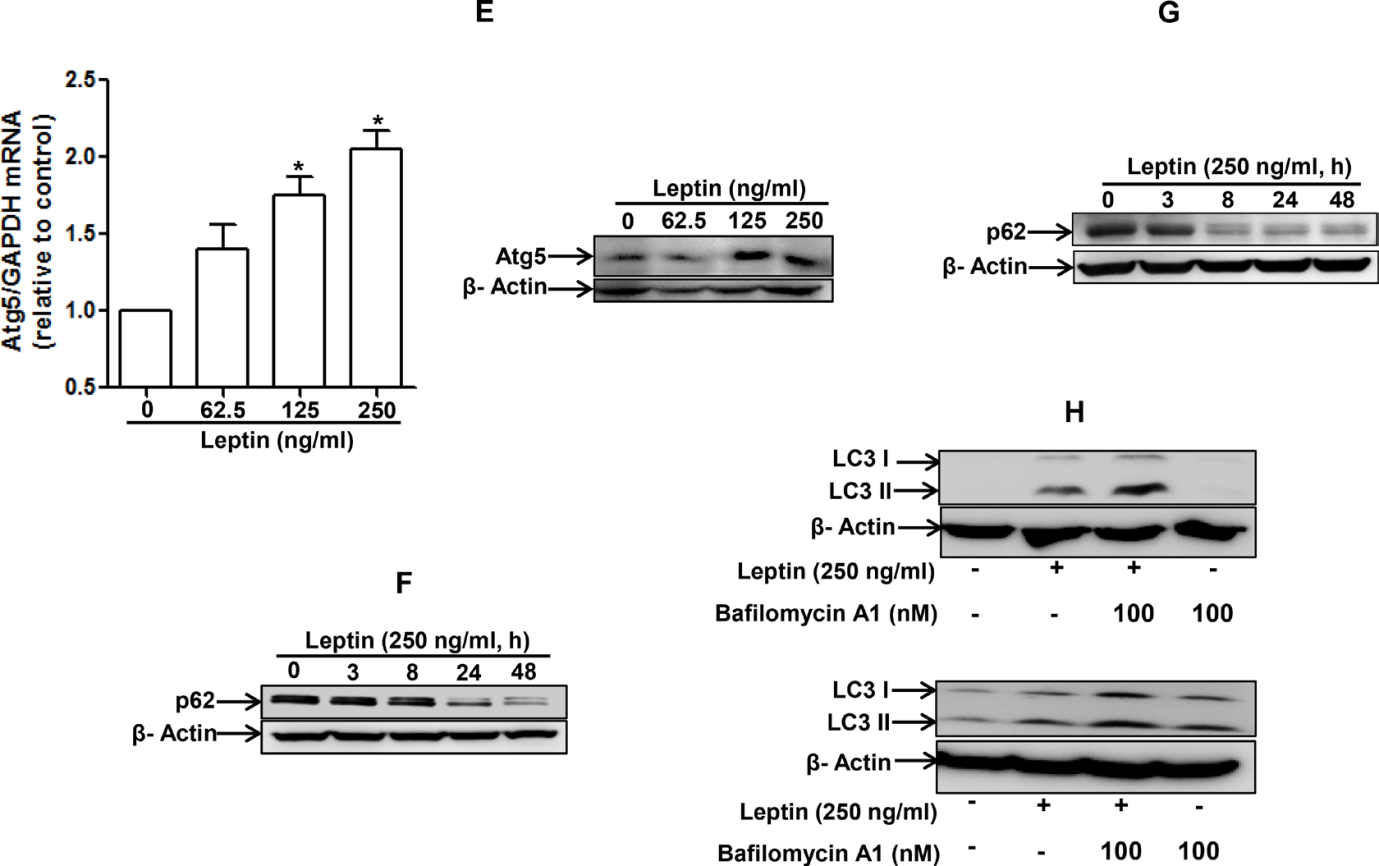

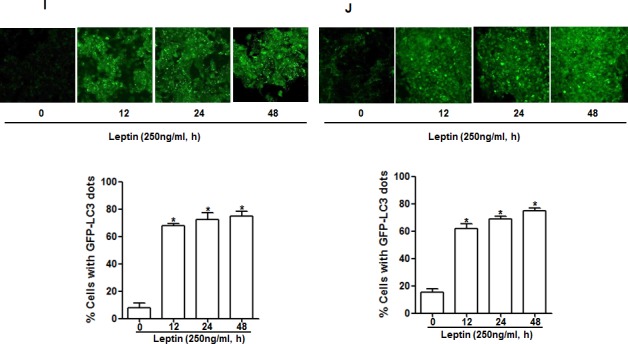
Effects of leptin on the expression of autophagy-related proteins (A) and (B) Effect of leptin on LC3B mRNA and LC3 II protein expression. HepG2 cells (A) and MCF-7 (B) cells were incubated with indicated leptin concentration for 48 h or leptin (250 ng/ml) for different time periods. LC3B mRNA expression level was determined by qRT-PCR as described previously (left panel). LC3 II protein expression level was determined by Western blot analysis as described previously. Representative images from three independent experiments that showed similar results are shown along with β-actin as an internal loading control. (C) and (D) Effect of leptin on beclin-1 mRNA and protein expression. HepG2 cells (C) and MCF-7 Cells (D) were incubated with the indicated concentration of leptin for 48 h.Beclin-1 mRNA expression level was determined by qRT-PCR (left panel) and beclin-1 protein expression level (right panel) was determined by Western blot analysis as described previously.(E) Effect of leptin on Atg5 expression. HepG2 cells were incubated with indicated leptin concentration for 48 h.Atg5 mRNA (left panel) and protein expression (right panel) was determined as described previously. (F) and (G) Effect of leptin on p62 expression. HepG2 cells (F) and MCF-7 cells (G) were treated with leptin (250ng/ml) for the indicated time points. p62 protein expression level was determined by Western blot analysis as described previously.(H) HepG2 (left panel) and MCF-7(right panel) cells were pretreated with Bafilomycin A1 for 2 h, followed by leptin treatment for 48 h.LC3 II proteins were determined by Western blot analysis. Representative image from three independent experiments is shown. (I) and (J) Effect of leptin on autophagosome formation. HepG2 cells (I) and MCF-7 cells (J) were transfected with eGFP-LC3 expression plasmid for 36 h, followed by treatment with leptin (250ng/ml) for the indicated time points. Autophagosome formation (LC3 dots) was measured by capturing the images using A1 Confocal Laser Microscope. Representative images from three independent experiments are shown along with quantitation of LC3 dots on the lower panel. Values are expressed as percentage cells with GFP-LC3. *P<0.05 compared with cells not treated with leptin.

To test our hypothesis that leptin causes growth of cancer cells through autophagy induction, we first assessed the effect of leptin on autophagy induction. As shown in Fig. 2A, leptin increased LC3B expression both at mRNA (left panel) and protein levels (right panel) in a time- and dose-dependent manner in HepG2 cells. Leptin treatment also produced the same effects on LC3 II protein expression in MCF-7 cells (Fig. 2B). Next, we examined the effect on the expression of beclin-1, which is essential for the initial stage of autophagic process. As depicted in Fig. 2C and 2D, leptin increased beclin-1 mRNA (HepG2) and protein expression in a dose-dependent manner both in HepG2 cells and MCF-7 cells. Similarly, leptin increased expression of Atg5, an essential Atg for the elongation of autophagosomes, both at mRNA and protein levels in HepG2 cells (Fig. 2E). In addition to expression of proteins related with autophagy, leptin treatment caused decrease in the level of p62 (Fig. 2F and 2G), indicating a possibility of enhancement of autophagy flux. To further confirm autophagy flux inducing effect of leptin, cells were pretreated with Bafilomycin A1, a selective autophagosome-lysosome inhibitor, followed by stimulation with leptin. In these experiments, we observed further increment in LC3 II protein levels both in HepG2 (upper panel) and MCF-7 (lower panel) cells (Fig. 2H), strongly suggesting autophagy flux inducing effect of leptin. Finally, we analyzed the effect on autophagosome formation via confocal microscopy in cells transfected with eGFP-LC3 plasmid. As shown in Fig. 2I and 2J, leptin significantly increased LC3 dots formation in both cell lines, confirming the autophagy inducing effect of leptin. Taken together, these data indicate that leptin treatment stimulates various characteristics of autophagy in cancer cells.

### Leptin-induced autophagy correlates to the suppression of apoptosis and growth of cancer cells

**Figure 3:**
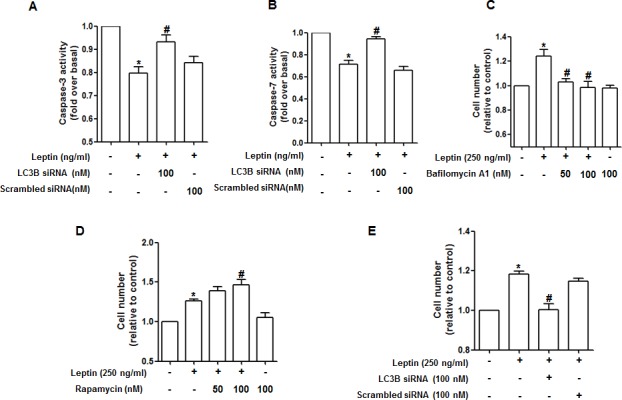
Role of autophagy induction in suppression of caspase-3 activity by leptin (A) and (B) HepG2 cells (A) and MCF-7 cells (B) were transfected with siRNA targeting LC3B or scrambled control, followed by incubation with indicated leptin concentration for 48 h.Caspase-3 activity for HepG2 cells or Caspase-7 activity for MCF-7 cells were determined as described previously. Values are presented as mean ± SEM (n=3). *P<0.05 compared to the cells not treated with leptin; #P< 0.05 compared with cells treated with leptin but not transfected with LC3B or scrambled control siRNA. (C) and (D) HepG2 cells were pretreated with either bafilomycin (C) or rapamycin (D) for 2 h, followed by treatment with leptin for 48 h. Cell viability was assessed by MTS assay as described previously. Values are presented as mean ± SEM (n=3). *P<0.05 compared with cells not treated with leptin; #P< 0.05 compared with cells treated with leptin. (E) Cells were transfected with siRNA targeting LC3B or scrambled control siRNA for 48 h. Cells were then treated with indicated concentration of leptin for 48 h and then cell number was measured by MTS assay as described previously. Values represent fold increase in comparison to the cells not treated with leptin and are expressed as mean ± SEM (n=3). *P<0.05 compared to the cells not treated with leptin; #P< 0.05 compared with cells treated with leptin but not transfected with LC3B or scrambled control siRNA.

Leptin suppresses apoptosis of cancer cells to induce tumor growth [7]. Autophagy and apoptosis negatively regulate each other in various experimental conditions [17]. To clarify the mechanisms for leptin-induced growth of cancer cells, we investigated the role of autophagy in modulation of apoptosis. As shown in Fig. 3A, leptin treatment suppressed caspase-3 activity in HepG2 cells consistent with previous reports. Interestingly, the suppressing effect on caspase-3 activity by leptin was significantly restored by LC3B gene silencing. The similar effects on caspase-7 activity were observed in MCF-7 cells (Fig. 3B), providing an evidence that autophagy activation plays a critical role in suppression of apoptosis by leptin. Gene silencing of Atg5 also produced similar effects on the modulation of caspase-3 activity in HepG2 cells (Supplementary Figure 1), confirming the role of autophagy in leptin-induced suppression of apoptosis. The role of autophagy induction in cancer growth was confirmed by cell viability/number assay. Leptin treatment induced increase in number of HepG2 and MCF-7 cells as expected. But, pretreatment with Bafilomycin A1, a selective autophagosome-lysosome inhibitor and inhibits late phase of autophagy, significantly suppressed leptin-induced growth of HepG2 (Fig. 3C), whereas pretreatment of cells with rapamycin, an inhibitor of mammalian target of rapamycin (mTOR) and acts as an autophagy activator, further increased cell number in leptin-treated cells (Fig. 3D). Finally, the role of autophagy induction in leptin-induced cancer cell growth was confirmed by gene silencing of LC3B. As indicated in Fig. 3E, knocking down of LC3B gene significantly suppressed leptin-induced increase in cell number. Gene silencing of Atg5 also prevented leptin-induced increase in cell number (Supplementary Figure 2), similar to the modulation of capase-3 activity. Taken together, all these results provide evidence that autophagy activation plays a critical role in leptin-induced cancer cell growth via suppression of apoptosis.

### Leptin-induced autophagy activation suppresses Bax protein expression

**Figure 4:**
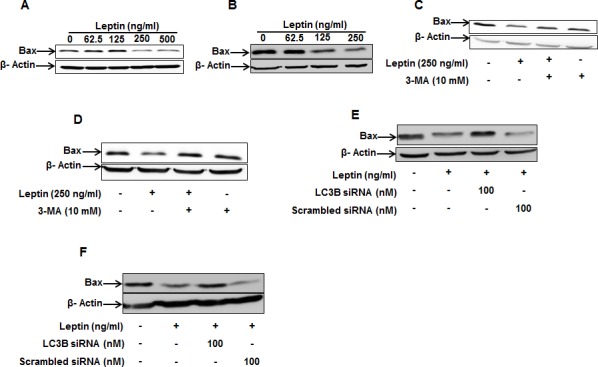
Role of autophagy induction in leptin-suppressed Bax expression (A) and (B) HepG2 cells (A) and MCF-7 Cells (B) were treated with indicated leptin concentration for 48 h and Bax protein expression levels were determined as described in materials and methods. (C) and (D) HepG2 cells (C) and MCF-7 cells (D) were pretreated with 3-MA for 2 h, followed by further stimulation with indicated concentration of leptin for 48h. Bax protein expression levels were determined by Western blot analysis as described previously. (E) and (F) HepG2 Cells (E) and MCF-7 cells (F) were transfected with siRNA targeting LC3B or scrambled control siRNA for 48 h. Cells were then treated with indicated concentration of leptin for 48 h. Bax protein levels were determined as described previously.

To further elucidate the molecular mechanisms underlying modulation of apoptosis by autophagy, we investigated whether autophagic process regulates apoptotic pathway by targeting Bax protein in leptin-treated cancer cells. As shown in Fig. 4, leptin treatment decreased Bax protein levels both in HepG2 (Fig. 4A) and MCF-7 cell lines (Fig. 4B). Next, to verify the functional role of autophagy in the suppression of Bax expression, cells were pretreated with 3-methyl adenine (3-MA). In these experiments, we observed that pretreatment with 3-MA restored expression level of Bax in HepG2 (Fig. 4C) and MCF-7 cells (Fig. 4D), indicating a crucial role of autophagy in suppression of Bax expression by leptin. Similar results were also observed by gene silencing of LC3B. As shown in Fig. 4E and 4F, LC3B silencing restored leptin-suppressed Bax protein levels in both cell lines.

### p53/FoxO3A signaling is implicated in autophagy induction by leptin in cancer cells

**Figure 5:**
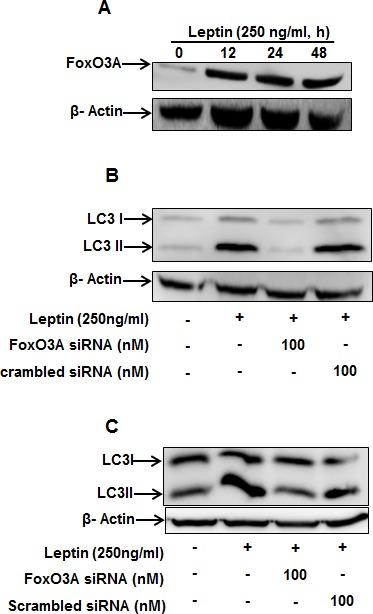
Role of FoxO3A signaling in activation of autophagic process by leptin (A) HepG2 cells were incubated with leptin for the indicated time points. FoxO3A protein expression levels were determined by Western blot analysis as described previously. (B) and (C) HepG2 (B) and MCF-7 (C) cells were transfected with FoxO3A siRNA for 48 h, followed by incubation with indicated concentration of leptin for 48 h. LC3 II protein expression levels were determined by Western blot analysis.

We next further explored the potential signaling mechanisms involved in leptin-induced autophagy induction. For this, we first examined the effect of leptin on FoxO3A expression, since FoxO3A is closely associated with expression of genes related with autophagy [23]. As depicted in Fig. 5, leptin treatment increased FoxO3A protein expression in a time-dependent manner (Fig. 5A) and FoxO3A gene silencing inhibited leptin-induced LC3II protein expression both in HepG2 (Fig. 5B) and MCF-7 cells (Fig. 5C), suggesting that FoxO3A signaling is implicated in leptin-induced autophagy in cancer cells.

**Figure 6:**
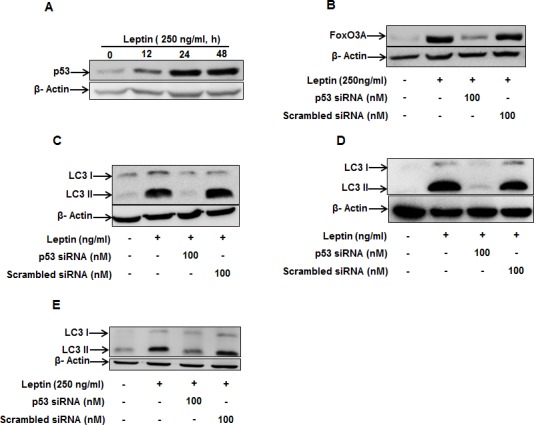
Role of p53 in the modulation of LC3 II protein expression by leptin (A) HepG2 cells were treated with leptin for the indicated time periods and p53 protein expression levels were determined by Western blot analysis as described previously. (B) HepG2 cells were transfected with siRNA targeting p53 or scrambled control siRNA for 48 h, followed by stimulation with leptin for additional 48 h. FoxO3A protein expression levels were determined by Western blot analysis as described previously. (C) and (D) HepG2 cells(C) MCF-7 cells (D) and HCT 116 cells(E) were transfected with siRNA targeting p53 or scrambled control siRNA. After 48 h incubation, cells were stimulated with leptin for 48 h. LC3 II protein expression levels were determined by Western blot analysis as described previously. Representative image from three independent experiments are shown.

For further characterization of the upstream signaling molecules, we investigated the involvement of p53. As shown in Fig. 6A, leptin increased p53 protein expression in a time-dependent manner in HepG2 cells. In addition, leptin-induced FoxO3A expression was prevented by gene silencing of p53 (Fig. 6B), providing evidence that p53 could regulate FoxO3A expression in leptin-treated cells. Furthermore, gene silencing of p53 completely abrogated leptin-induced LC3II protein expression both in HepG2 (Fig. 6C), MCF-7 cells (Fig. 6D) and colon cancer HCT 116 cells (Fig. 6E), suggesting a critical role of p53 signaling in leptin-induced autophagy induction.

### Leptin induces tumor growth in HepG2 tumor xenografts model via autophagy induction

**Figure 7:**
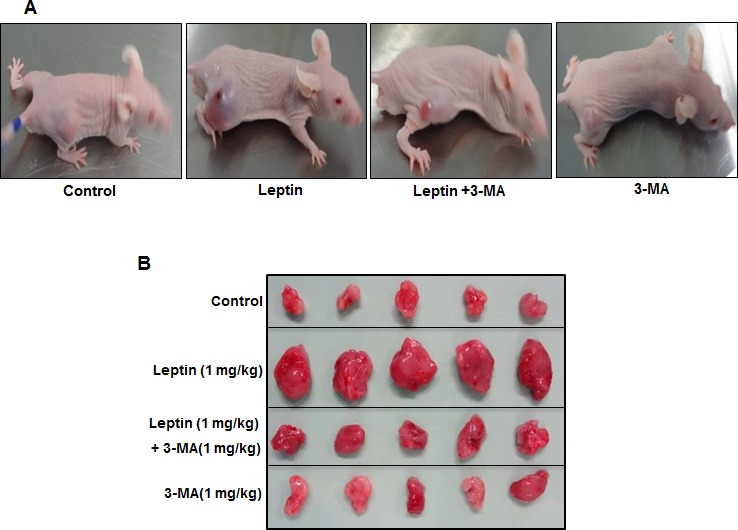

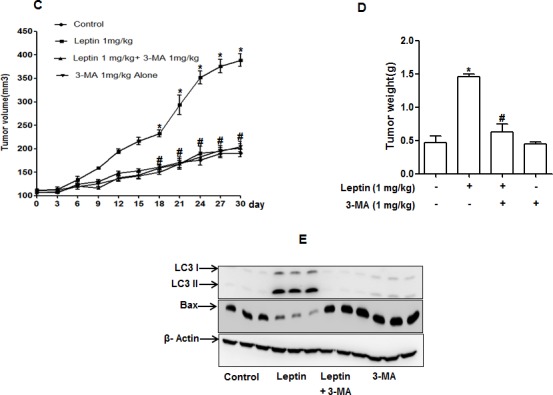
Role of leptin-induced autophagy in tumor growth in xenograft model HepG2 cells were injected subcutaneously into the rear flanks of 4 weeks old male BALB/c nude mice. After two weeks, animals were randomly divided into four groups; Control, Leptin (1 mg/kg), Leptin (1 mg/kg) combined with 3-MA (1 mg/kg) and 3-MA (1 mg/kg) alone. Leptin and 3-MA were given intraperitoneally every 36 h for 4 weeks. (A) Representative mice from each group at the end of the treatment. (B) Animals were sacrificed and tumor tissues were collected at the end of treatment. (C) (Tumor volume was measured twice weekly as described in materials and methods. D) Tumor tissues were isolated from each mouse after sacrifice and weight was measured. Values are expressed as mean ± SEM (n=5). *P<0.05 compared to control mice; #P< 0.05 compared to mice treated with leptin.

While autophagy induction correlates to the survival of cancer cells, the relationship if any between leptin-induced tumor growth and autophagy activation has not been explored. In this study, we hypothesized that leptin-induced tumor growth could be mediated by autophagy activation. For confirmation of the results observed from *in vitro* experiments, we prepared HepG2 tumor xenografts in BALB/c nude mice and confirmed these results in *in vivo* model. We first investigated the effect of leptin on tumor growth in. As shown in Fig. 7A and 7B, intraperitoneal injection with leptin promoted tumor growth in xenograft model consistent with the previous reports, also evidenced by increase in tumor volume (Fig. 7C) and tumor weight (Fig. 7D). Importantly, co-treatment with 3-MA, a pharmacological inhibitor of type III PI3K and finally inhibits autophagy, prevented leptin-induced tumor growth without significant effect by treatment with 3-MA alone, indicating a critical role of autophagic process in leptin-induced tumor growth. In xenograft model implanted with HepG2 cells, leptin treatment significantly increased expression of LC3II protein in tumor tissues, whereas 3-MA treatment inhibited leptin-induced LC3II protein expression (Fig. 7E, upper panel). Furthermore, suppression of Bax expression was almost completely recovered by co-administration with 3-MA (Fig. 7E, lower panel). These results further substantiate autophagy induction by leptin *in vivo*, and its role in tumor growth and suppression of apoptosis.

## DISCUSSION

Recently there has been increasing evidence demonstrating the close relationship of obesity with increased incidence of cancers, particularly those of breast, liver and colon cancers [5]. Various mechanisms have been proposed to elucidate the correlation between obesity and cancer development. One of them is alteration of adipokines level in obese people. For example, blood leptin level is proportional to the obese, but adiponectin level is decreased in the obese. Alterations in production of adipokines, and the resultant metabolic or endocrine effects are thought to play a crucial role in development of liver and breast cancer [24-26]. In particular, enhanced serum leptin levels could play an integral role in cancer incidence as well as progression. Therefore, leptin is considered as one of the target molecules linking obesity and higher risk of cancer development.

Autophagy plays a critical role in a number of biological responses, such as modulation of metabolic functions and cancer development. A recent article has reported that administration of leptin stimulates autophagy in HeLa cells [27] and suggests a possible role of autophagy in controlling the neuroendocrine function of leptin. However, indeed, this study also showed that genetic inactivation of leptin/leptin receptor system induces autophagic process in various tissues, indicating a complicated response of leptin on autophagy induction. In addition, the effect of leptin on autophagy induction in different types of cancer cells and its role in leptin-induced various biological responses have not been explored. Leptin has been shown to utilize various signaling pathways, including JAK/STAT, MAPKs and PI3K. However, the detailed molecular mechanisms underlying leptin-induced tumor growth are not clearly understood. In the present study, we observed that leptin treatment induced increase in expression of genes-related with autophagy, including beclin-1, Atg5 and LC3II, induced autophagosome formation, and prevented accumulation of p62 both in HepG2 and MCF-7 cells (Fig. 2). All these results verified the autophagy inducing effect of leptin that could impact various biological responses attributed to leptin. Furthermore, we demonstrated that leptin treatment causes activation of autophagic process via p53/FoxO3A axis and autophagy activation plays a critical role in leptin-induced tumor growth both *in vivo* and *in vitro* model

Autophagy was originally reported as a different type of cell death from apoptosis [28] and thus regarded to serve as an anti-tumor mechanism. However, the exact role of autophagy in cancer is controversial and recent studies have revealed that autophagy also functions as a survival mechanism in cancer cells against cellular stress [29], indicating that the role of autophagy in cancer development would be context-dependent. For example, mutation of Beclin-1 gene increases the frequency of malignancies in hepatitis B virus-induced premalignant injury [30]. On the other hand, deletion of Beclin-1 results in tumor cell death in hypoxic regions [31]. Even if detailed mechanisms underlying determination of the role of autophagy in the fate of cancer is not clearly understood, it is generally accepted that autophagic process prevents cancer development in the initial stage (or healthy tissue) via preventing the accumulation of dysfunctional and mutated cellular components, while autophagy promotes tumorigenesis at the late stage of tumor via protection of cancer cells and generates resistance to the treatment of chemotherapeutic agents [16]. Although autophagy has dual role in cancer development, recent studies have highlighted that autophagy contributes to the development of cancer and acts as a survival mechanism in cancer cells. It has been also shown that autophagy induces cancer development via suppression of apoptotic process. Accumulating evidences suggest crosstalk between autophagy-related proteins such as Atg5, Beclin-1, LC3B and apoptotic proteins such as Bax, Calpain, and Caspases that ultimately determines the fate of the cells [17]. For example, Bcl-2 family proteins such as Bcl-2, Bcl-xL and Mcl-1, interacts with Beclin-1 through BH3 domain of Beclin-1, resulting in autophagy inhibition [32]. Autophagy also targets apoptosis-related proteins such as Bax for degradation, and cleaves caspases, thereby inhibiting apoptosis [33]. Leptin has been shown to induce proliferation of hepatocellular [7], esophageal [3], breast [34], prostate [9], colon [35], and gastric cancer cell lines [36] and suppresses apoptosis in hepatocellular carcinoma cell lines [7] and esophageal adenocarcinoma cells [3] etc. Although previous studies have demonstrated mutual negative relationship between autophagy and apoptosis, the role of leptin-induced autophagy in the suppression of apoptosis in cancer cells has not been reported. Data presented in this study clearly demonstrate for the first time that leptin-induced autophagic process plays an important role in tumor growth via attenuation of apoptosis (Fig. 3, 4 and Supplementary Figures).

**Figure 8:**
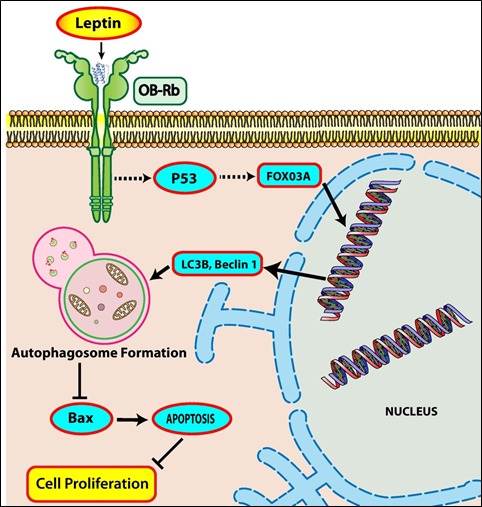
Proposed model for autophagy induction by leptin and its role in suppression of apoptosis in cancer cells Leptin treatment causes cancer cell growth via stimulating various signaling pathways. Cell growth stimulation could manifest in conditions where cell survival is enhanced and cell death is inhibited. Autophagy activation is one of the important survival mechanisms in the face of stressful conditions, such as that occur during tumor progression. Leptin increases expression of autophagy related genes, including beclin-1 and LC3B, as well as induces autophagy flux, as evidenced by decrease in p62 protein accumulation, in cancer cells *in vitro* and nude mice *in vivo*. In addition, leptin treatment suppresses expression of Bax, an important apoptotic protein, thereby inhibiting apoptosis in both HepG2 and MCF-7 cells, and *in vivo*. Apoptosis and autophagy have been regarded to negatively modulate each other's action. Once autophagic process is activated by leptin treatment, it suppresses apoptosis of cancer cells via degradation of Bax. Leptin treatment also increases p53 and FoxO3A protein expression, and intriguingly p53 activation regulates FoxO3A expression. FoxO3A in turn increases expression of genes involved in autophagy, implying that p53/FoxO3A axis plays a critical role in leptin-induced increase in autophagy induction, and this mediates cell growth in both hepatic and breast cancer cells, and tumor growth in vivo. Further studies to decipher detailed molecular mechanisms underlying leptin-induced p53 and regulation of FoxO3A by p53 need to be carried out.

In addition to the regulation of apoptosis, we also found that leptin induces cell-cycle progression in HepG2 cells and intriguingly, leptin-induced autophagy also plays an important role in cell cycle progression by leptin (unpublished observations). Based on these results, autophagy seems to play a crucial role in leptin-induced tumor growth via stimulating cell cycle, as well as regulating apoptotic process. At this stage, we could not identify the mechanisms underlying the modulation of cell cycle progression by autophagy activation. Future studies may unravel molecular links between autophagy activation and cell cycle progression by leptin.

p53, originally known as a tumor suppressor protein, has been also shown to influence autophagy. The effect of p53 on autophagy modulation is controversial as both pro-autophagic and anti-autophagic functions could be manifested by p53 based on its subcellular localization [18]. The cytoplasmic localization of p53 has been shown to inhibit autophagy via interaction with the autophagy-related molecules and suppressing the initial steps of autophagic flux involving ULK1 [37]. In contrast, nuclear localization of p53 generates pro-autophagic effects mostly via regulation of mTOR, which is considered as one of the key signaling pathways leading to autophagy activation [18, 38]. On this basis, it has been proposed that p53 modulates autophagy in a differential manner and plays a role in maintaining autophagic homeostasis. In the present study, we observed that leptin induces autophagy induction via p53 expression, rather than modulation of subcellular localization. The effect of leptin on p53 expression is a matter of intense debate. Nkhata et al., has reported that leptin significantly increases p53 protein expression in MCF-7 cells [39], while other reports have suggested that leptin decreases p53 expression, thereby promoting cell proliferation [40], showing that effect of leptin on p53 expression and its role in cell death/survival are not conclusive. In the present study, we demonstrated that leptin increases p53 expression, which is implicated in autophagic process via induction of FoxO3A and autophagy related genes both in HepG2 and MCF-7 cells. To the best of our knowledge, this is the first report regarding the activation of p53 and its crucial role in autophagy activation by leptin.

The *forkhead-box* (Fox) family of transcription factors plays an evolutionarily conserved role in metabolism, differentiation and proliferation/arrest of the cells [41]. In particular, FoxO3A is reported as a potent transcriptional activator responsible for induction of autophagy-related genes [42, 43]. Recent data also indicate that FoxO3A plays a pivotal part in the regulatory network that controls autophagy and cancer [44]. FoxO3A activity is basically controlled by its subcellular localization or changes in transcriptional activity mostly regulated by various types of kinases [45]. For example, direct phosphorylation of FoxO3A by AKT causes export from nucleus, therefore transcription of target genes by FoxO3A is suppressed, while phosphorylation by JNK promotes nuclear localization [46]. On the other hand, AMPK enhances transcriptional activity of FoxO3A via direct phosphorylation of FoxO3A [41] or promoting SIRT1-induced deacetylation [46]. In the present study, we found that leptin modulates FoxO3A activity via enhancing protein expression level of FoxO3A, rather than regulation of activity. FoxO3A protein expression level is also regulated by various mechanisms, including modulation of protein stability and degradation, and at the transcription level [47, 48]. Previous studies have demonstrated that p53 transactivates FoxO3A by binding in the second intron region of FoxO3A in fibroblasts and thymocytes [49]. In the present study, while we clearly demonstrated that p53 is implicated in leptin-induced increase in FoxO3A expression in hepatic cancer cells, detailed mechanisms underlying are not clearly understood and additional studies are required to elucidate the interaction between p53 and FoxO3A in leptin-treated cancer cells. With regards to the modulation of tumor growth, interestingly, FoxO3A behaves as a Janus-faced protein. While FoxO3A is widely known to cause tumor suppression and therefore is considered as a promising target for the treatment of cancer [41], recent studies have also indicated that FoxO3A is also involved in tumor progression. For example, FoxO3A acts as a transcription factor leading to the expression of anti-apoptotic kinase, homeodomain-interacting protein kinase 3 (HIPK3), via coordinated action with β-catenin, and confers resistance to apoptosis [50]. Data presented in this study demonstrates that FoxO3A expression and subsequent autophagy induction by leptin treatment leads to the suppression of apoptosis.

In addition to the proliferation of cancer cells, autophagy activation has been shown to cause invasion of hepatocellular carcinoma cells by enhancing MMP-9 expression [51] and glioblastoma cells by regulating DRAM1 and p62 [52]. Leptin treatment also showed increase in MMP-9 expression in hepatic cancer cells [53] and endothelial cells [54] to induce invasion of tumor cells. Furthermore, FoxO3A activation has been also shown to cause tumor progression through induction of matrix metalloproteinase, such as MMP-9 and MMP-13 [55]. Based on previous reports, it would be possible that autophagy induction plays a role in leptin-induced tumor invasion and this hypothesis remains to be further investigated. Taken together, data in the present study demonstrate the first evidence for the involvement of p53-FoxO3A signaling in leptin-induced autophagy activation. Intriguingly, leptin seems not to be causing autophagy activation in MDA-MB-231 breast cancer cell which has mutant p53 in our experimental conditions, as evidenced by no significant change in LC3 II protein expression levels (Supplementary Figure 4). Future studies may provide further insights into the mechanisms underlying role of p53-FoxO3A axis in autophagic responses and many other pathophysiological responses induced by leptin.

Among various types of cancer, hepatic and breast cancer are considered that their developments are closely related with obesity. The present study suggests that hepatic and breast cancer can be highly developed in the obese due to leptin-induced autophagic process. Besides leptin, the circulating level of adiponectin is also altered in obese people. Decreased adiponectin level in the obese is associated with cancer development [24]. We also observed that adiponectin treatment induces autophagy in hepatic cancer cells. Furthermore, inhibition of autophagy enhanced caspase-3 activity and cytotoxic effect by adiponectin (In Press), suggesting that autophagy plays a regulatory role in cancer cell death induced by adiponectin. While it is widely known that alterations in adipokines level are closely related with cancer development, the molecular mechanisms are largely unknown. The results observed in this study and previous reports suggest that autophagy plays a critical role in modulation of tumor growth by adipokines. Since autophagy renders a survival advantage in certain cancer types/conditions, inhibitors of autophagy are being investigated in both pre-clinical and clinical studies for enhancing therapeutic efficacy via sensitizing tumor cells to the conventional chemotherapies [56]. The current study also suggests that modulation of autophagy would be a novel strategy for the prevention or treatment for obesity-induced cancer. Many previous studies have used leptin concentration ranging from 10-200 nM, which is equivalent to 160-3200 ng/ml. In fact, these concentrations are higher than that seen in normal subjects, but commonly observed in obese subjects [3, 7, 9, 34, 57]. The leptin concentration used in our study would be relevant to the obese people and was within the range used by many previous studies. In addition, we also found that leptin enhanced cell number and suppressed caspase-3 activity at 125 ng/ml was significantly restored by autophagy inhibition (LC3B silencing) in HepG2 cells suggesting the critical role of autophagy activation in the suppression of apoptosis and increase in cell number by treatment with lower concentration of leptin too (Supplementary Figure 3).

In conclusion, the present study has demonstrated that leptin induces autophagy in hepatic and breast cancer cells, which in turn leads to the suppression of apoptosis. Moreover, p53 and FoxO3A signaling mediates leptin-induced autophagy activation (Fig. 6). Leptin being a pleiotropic adipokine has been shown to cause activation of various signaling pathways leading to tumor growth/cell proliferation, such as via up regulation of angiogenesis in breast cancer [58], or suppression of apoptosis in colon cancer cells [59], and hepatic cancer cells [7]. In the present study, we have further demonstrated that autophagy induction play an important role in leptin-induced suppression of apoptosis via degradation of Bax, thereby increasing cell number in HepG2 and MCF-7 cells, and tumor growth in tumor xenograft model in BALB/C nude mice *in vivo* implanted with HepG2 cells. Therefore, the present study has further identified that autophagy activation would be a novel mechanism underlying leptin-induced tumor growth. In obese individuals, serum leptin levels are higher and regarded to have positive association with cancer progression. Based on our results, inhibition of autophagy could impact leptin-induced tumor progression and further provoke new therapeutic strategies in obesity-associated cancer development.

## MATERIALS AND METHODS

### Materials

All the cell culture reagents were obtained from Hyclone laboratories (South Logan, Utah, USA). Recombinant mouse leptin was purchased from Sigma-Aldrich (St Louis, MO, USA). Cell proliferation and Caspase-3 activity assay kits were procured from Promega Corporation (Madison, WI, USA). Antibody against beclin-1, LC3II, p62, Bax, p53 and FoxO3A were purchased from Cell Signaling Technology Inc. (Beverly, MA, USA). Antibody against Atg5 was obtained from Thermo Scientific Inc (Rockford, IL, USA). 3-Methyl Adenine (3–MA) was purchased from Tocris Bioscience (Bristol, UK). All other chemicals were procured from Sigma-Aldrich unless mentioned elsewhere.

### HepG2 and MCF-7 cell culture

HepG2 and MCF-7 cell lines were purchased from American Type Culture Collection (ATCC, Rockville, MD, USA) and routinely cultured in Dulbecco's Modified Eagle Medium (DMEM) supplemented with 10% FBS and 1% penicillin-streptomycin in the presence or absence of 0.1% amphotericin, respectively.

### Cell viability assay (MTS assay)

For the determination of cell number, MTS assay was performed essentially as described previously [60]. Cell viability/number was determined via microplate reader (Molecular Devices, CA, USA) by measuring absorbance at 490 nm.

### Caspase-3/7 activity assay

Caspase-3/7 activity was assessed by using Caspase-Glo 3/7 assay kits (Promega Corporation, Madison, USA) essentially as described previously [60]. Caspase-3 or caspase-7 activity was assessed by measuring luminescence from the cleavage of luminogenic substrate Ac-DEVD-pNA with a micro-plate reader (Flurostar Optima, BMG Labtech, Germany).

### RNA isolation, reverse transcription (RT) and quantitative PCR (qPCR)

For the measurement of mRNA levels of genes of interest, total RNAs were isolated using Qiagen lysis solution (Qiagen, Maryland, USA) according to the manufacturer's instructions. cDNA was then synthesized via reverse transcription of 1 μg of total RNA. Real time-PCR amplification was then performed with a Roche Light Cycler 2.0 (Mannheim, Germany) using the absolute QPCR SYBR green capillary mix AB gene system (Thermo scientific, UK) essentially as described previously [23]. The primer sequences used for amplification of target human genes are listed in Table 1.

**Table 1:** Sequences of human primers used in quantitative RT-PCR

Target gene	Primer	Nucleotide sequence
GAPDH	F R	5′-ACCACAGTCCATGCCATCAC-3′ 5′-TCCACCACCCTGTTGCTGTA-3′
Beclin-1	F R	5′-CTTACCACAGCCCAGGCGAAAC-3′ 5′-GCCAGAGCATGGAGCAGCAA-3′
LC3B	F R	5′-ACCATGCCGTCGGAGAAG-3′ 5′-GGTTGGATGCTGCTCTCGAA-3′
Atg5	F R	5′-AACTGAAAGGGAAGCAGAACCA-3′ 5′-CCATTTCAGTGGTGTGCCTTC-3′

### Transient transfection with small interfering RNA (siRNA)

Cells were transfected with corresponding siRNA of target gene or scrambled control siRNA with Hiperfect transfection reagent (Qiagen, Hilden, Germany) according to the manufacturer's instructions followed by leptin treatment as indicated in figure legends. The efficiency of siRNA transfection was assessed by Western blot analysis after 48 h of transfection. The siRNA duplexes were procured from Bioneer (Daejeon, South Korea) and are listed in Table 2.

**Table 2:** Sequences of small interfering RNA used in transfection

Target gene	Primer	Nucleotide sequence
LC3B	F R	5′-GACUGUCUCGUUUAGACUG-3′ 5′-CAGUCUAAACGAGACAGUC-3′
p53	F R	5′-CACUACAACUACAUGUGUA-3′ 5′-UACACAUGUAGUUGUAGUG-3′
FoxO3A	F R	5′-GACGAUGAUGCGCCUCUCU-3′ 5′-AGAGAGGCGCAUCAUCGUC-3′
Scrambled Control	F R	5′-CCUACGCCACCAAUUUCGU-3′ 5′-ACGAAAUUGGUGGCGUAGG-3′

### Confocal microscopic analysis

For confocal microscopic analysis, HepG2 and MCF-7 cells were transfected with enhanced green fluorescent protein (eGFP)-LC3 expression plasmid using Fugene HD transfection reagent (Promega) according to the manufacturer's instructions essentially as described previously [23].

### Tumorigenecity assay in nude mice

All experiments were performed in accordance with guidelines issued by Yeungnam University Research Committee for the Care and Use of Laboratory Animals. For the establishment of xenografts, HepG2 cells (1 × 107 cells/200 μl) were injected subcutaneously into the rear flanks of 4 weeks old male BALB/c nude mice, obtained from Orient Ltd (Osan, South Korea). After two weeks of initial implantation, animals were randomly divided into four groups (n=5 per group) as mentioned in figure legends. Leptin and 3-MA were injected intraperitoneally every 36 h for the duration of treatment. Tumors were measured twice weekly using digital caliper, with tumor volume (V) calculated as, V = (width)^2^ × length/2. The animals were sacrificed after 4 weeks of treatment, and the primary tumor was excised, weighed, and subjected to further analysis.

### Preparation of cellular extracts, tumor lysates and Western blot analysis

To determine protein expression level of the genes of interest, cellular extracts were prepared essentially as described previously [23]. For the preparation of tumor lysates, about 100 mg tumor tissues were homogenized in cold PBS using Bio-homogenizer and centrifuged at 1500 g for 3 min. To remove blood and other debris, tissue pellets were again washed with PBS and centrifuged at 10 000 g for 10 min. After removal of supernatant, tissue pellets were subjected for lysis in RIPA buffer containing protease inhibitor and kept in ice for 2 h. Tissue samples were then stored at −20 °C overnight, followed by thawing and ultra-sonication using JAC 4020 ultrasonicator (KODO Ltd, South Korea) at 40 KHz for 10 min. Finally, the tissue pellets were centrifuged at 13 000 g for 15 min, and the supernatants containing solubilized protein were separated. For Western blot analysis, 30-50 μg of solubilized proteins were loaded, resolved by 10-15% SDS-PAGE and transferred to PVDF membranes. The membrane was incubated with the designated primary and secondary antibodies, and chemiluminescent images of the blots were captured using a Fujifilm LAS-4000 mini (Fujifilm, Tokyo, Japan). The membranes were then stripped and reprobed with antibody against β-actin used as the loading control.

### Statistical analysis

Values are presented as mean ± SEM of at least three independent experiments. Data were analyzed by one-way analysis of variance (ANOVA) followed by Tukey's multiple comparison tests using Graph Pad prism software version 5.01 (California, USA). Differences between groups were considered to be significant at p<0.05.

## SUPPLEMENTARY MATERIAL FIGURES


