# Correction: Autophagy induction by leptin contributes to suppression of apoptosis in cancer cells and xenograft model: Involvement of p53/FoxO3A axis

**DOI:** 10.18632/oncotarget.27467

**Published:** 2020-07-28

**Authors:** Saroj Nepal, Mi Jin Kim, Jin Tae Hong, Sang Hyun Kim, Dong-Hwan Sohn, Sung Hee Lee, Kyung Song, Dong Young Choi, Eung Seok Lee, Pil-Hoon Park

**Affiliations:** ^1^ College of Pharmacy, Yeungnam University, Gyeongsangbuk-do, Republic of Korea; ^2^ College of Pharmacy and Medical Research Center, Chungbuk National University, Cheongju, Chungbuk, Republic of Korea; ^3^ Department of Pharmacology, School of Medicine, Kyungpook National University, Daegu, Republic of Korea; ^4^ Institute of Pharmaceutical Research and Development, College of Pharmacy, Wonkwang University, Iksan, Jeonbuk, Republic of Korea

**This article has been corrected:** During figure processing, identical Western blot images were mistakenly placed in both Fig. 2E and 2F. The existing image in Figure 2F is correct; the image for 2E was the incorrect duplicate. The corrected Figure 2E, obtained using original data, is shown below. The authors declare that these corrections do not change the results or conclusions of this paper.

Original article: Oncotarget. 2015; 6:7166–7181. 7166-7181. https://doi.org/10.18632/oncotarget.3347

**Figure 2:**
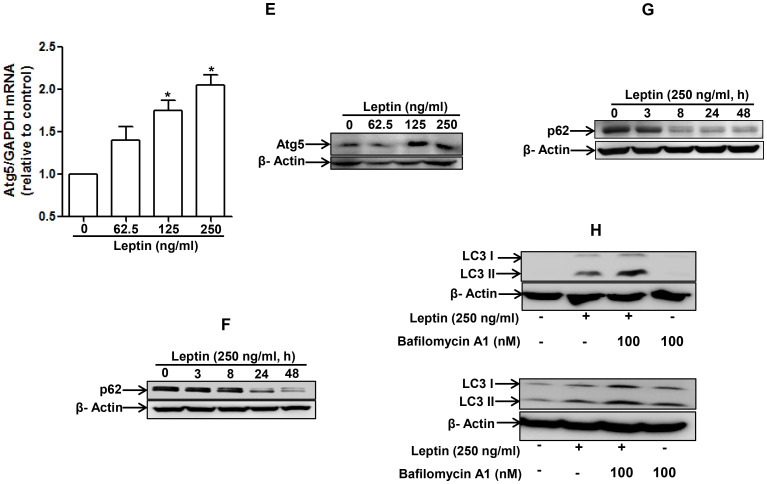
Effects of leptin on the expression of autophagy-related proteins. (**A**) and (**B**) Effect of leptin on LC3B mRNA and LC3 II protein expression. HepG2 cells (A) and MCF-7 (B) cells were incubated with indicated leptin concentration for 48 h or leptin (250 ng/ml) for different time periods. LC3B mRNA expression level was determined by qRT-PCR as described previously (left panel). LC3 II protein expression level was determined by Western blot analysis as described previously. Representative images from three independent experiments that showed similar results are shown along with β-actin as an internal loading control. (**C**) and (**D**) Effect of leptin on beclin-1 mRNA and protein expression. HepG2 cells (C) and MCF-7 Cells (D) were incubated with the indicated concentration of leptin for 48 h.Beclin-1 mRNA expression level was determined by qRT-PCR (left panel) and beclin-1 protein expression level (right panel) was determined by Western blot analysis as described previously. (**E**) Effect of leptin on Atg5 expression. HepG2 cells were incubated with indicated leptin concentration for 48 h.Atg5 mRNA (left panel) and protein expression (right panel) was determined as described previously. (**F**) and (**G**) Effect of leptin on p62 expression. HepG2 cells (F) and MCF-7 cells (G) were treated with leptin (250 ng/ml) for the indicated time points. p62 protein expression level was determined by Western blot analysis as described previously. (**H**) HepG2 (left panel) and MCF-7(right panel) cells were pretreated with Bafilomycin A1 for 2 h, followed by leptin treatment for 48 h.LC3 II proteins were determined by Western blot analysis. Representative image from three independent experiments is shown. (**I**) and (**J**) Effect of leptin on autophagosome formation. HepG2 cells (I) and MCF-7 cells (J) were transfected with eGFP-LC3 expression plasmid for 36 h, followed by treatment with leptin (250ng/ml) for the indicated time points. Autophagosome formation (LC3 dots) was measured by capturing the images using A1 Confocal Laser Microscope. Representative images from three independent experiments are shown along with quantitation of LC3 dots on the lower panel. Values are expressed as percentage cells with GFP-LC3. *P<0.05 compared with cells not treated with leptin.

