# Supplementary material for: Autophagy induction by leptin contributes to suppression of apoptosis in cancer cells and xenograft model: Involvement of p53/FoxO3A axis
Source: Oncotarget. 2015 Jan 31;6(9):7166–81. doi: 10.18632/oncotarget.3347 (PMC4466676; doi:10.18632/oncotarget.3347)
Supplement: Supplementary file 1 [file oncotarget-06-7166-s001.pdf]

Autophagy induction by leptin contributes to suppression of apoptosis in cancer cells and xenograft model: Involvement of p53/FoxO3A axis

Supplementary Material

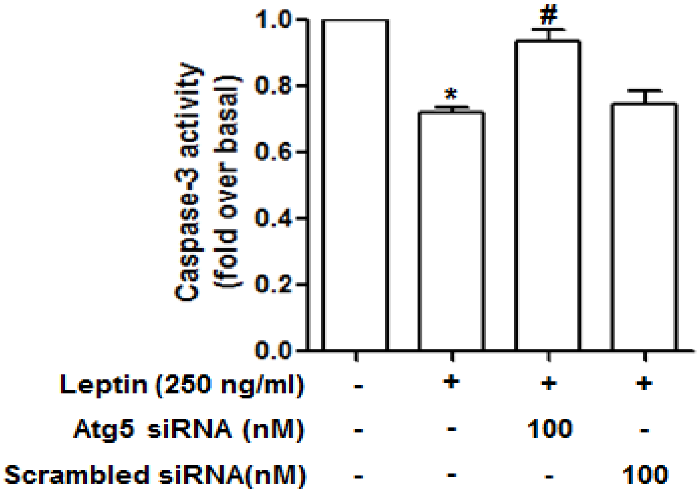

Supplementary figure 1: HepG2 cells were transfected with siRNA targeting Atg5 or scrambled control siRNA for 36 h and were incubated with leptin (250 ng/ml) for 48 h. Caspase-3 activity was determined as described previously. Values are presented as mean ± SEM (n=3). *P<0.05 compared to the cells not treated with leptin; $^{\#}$P< 0.05 compared with cells treated with leptin but not transfected with Atg5 or scrambled control siRNA.

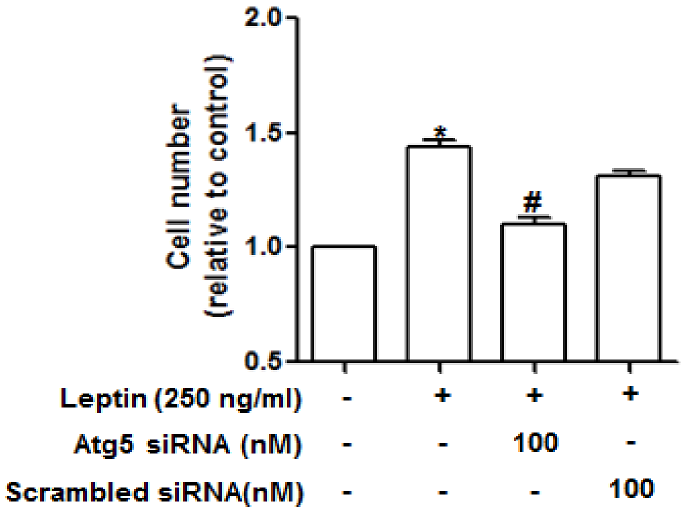

Supplementary figure 2: HepG2 cells were transfected with siRNA targeting Atg5 or scrambled control siRNA for 36 h and were stimulated with leptin (250 ng/ml) for 48 h. Cell number was determined by MTS assay as described previously. Values are presented as mean ± SEM (n=3). *P<0.05 compared to the cells not treated with leptin; $^{\#}$P< 0.05 compared with cells treated with leptin but not transfected with Atg5 or scrambled control siRNA.

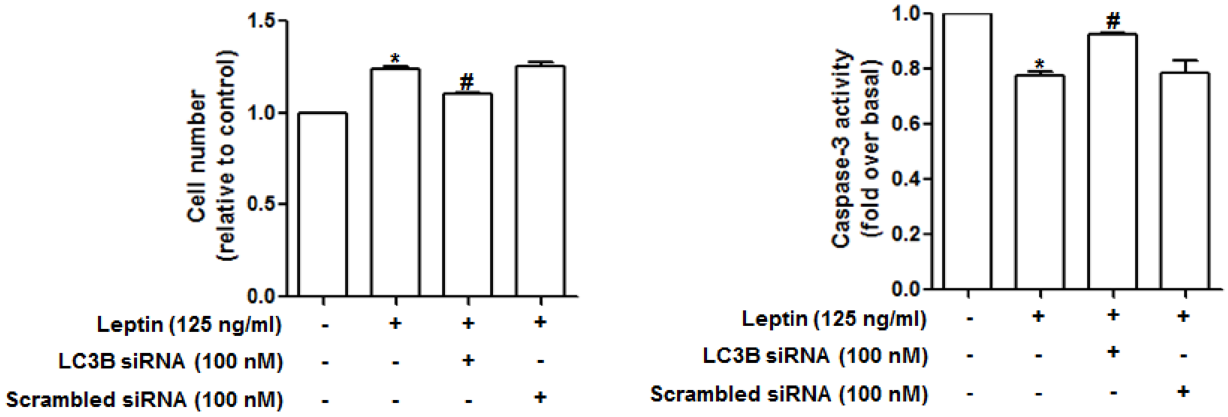

Supplementary figure 3: HepG2 cells were transfected with siRNA targeting LC3B or scrambled control, followed by incubation with indicated leptin concentration for 48 h. Cell number (Left panel) and caspase-3 activity were determined as described previously. Values are presented as mean ± SEM (n=3). *P<0.05 compared to the cells not treated with leptin; #P< 0.05 compared with cells treated with leptin but not transfected with LC3B or scrambled control siRNA.

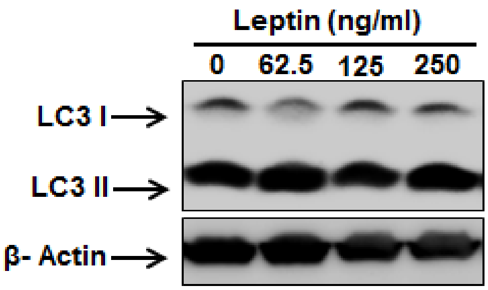

Supplementary figure 4: MDA-MB-231 cells were incubated with indicated leptin concentration for 48 h .LC3 II protein expression level was determined by Western blot analysis as described previously. Representative images from three independent experiments that showed similar results are shown along with β-actin as an internal loading control.
